# Heritable Influence of DBH on Adrenergic and Renal Function: Twin and Disease Studies

**DOI:** 10.1371/journal.pone.0082956

**Published:** 2013-12-31

**Authors:** Dalal N. Pasha, Jason T. Davis, Fangwen Rao, Yuqing Chen, Gen Wen, Maple M. Fung, Manjula Mahata, Kuixing Zhang, Danuta Trzebinska, Maja Mustapic, C. Makena Hightower, Michael S. Lipkowitz, Ming Ji, Michael G. Ziegler, Caroline M. Nievergelt, Daniel T. O'Connor

**Affiliations:** 1 Department of Medicine, University of California at San Diego, La Jolla, California, United States of America; 2 Department of Pharmacology, University of California at San Diego, La Jolla, California, United States of America; 3 Department of Psychiatry, University of California at San Diego, La Jolla, California, United States of America; 4 Graduate School of Public Health, Division of Epidemiology and Biostatistics, San Diego State University, San Diego, California, United States of America; 5 Veterans Affairs San Diego Healthcare System, La Jolla, California, United States of America; 6 Department of Medicine, Georgetown University, Washington, District of Columbia, United States of America; University Medical Center Utrecht, Netherlands

## Abstract

**Background:**

Elevated sympathetic activity is associated with kidney dysfunction. Here we used twin pairs to probe heritability of GFR and its genetic covariance with other traits.

**Methods:**

We evaluated renal and adrenergic phenotypes in twins. GFR was estimated by CKD-EPI algorithm. Heritability and genetic covariance of eGFR and associated risk traits were estimated by variance-components. Meta-analysis probed reproducibility of DBH genetic effects. Effect of *DBH* genetic variation on renal disease was tested in the NIDDK-AASK cohort.

**Results:**

Norepinephrine secretion rose across eGFR tertiles while eGFR fell (p<0.0001). eGFR was heritable, at h^2^ = 67.3±4.7% (p = 3.0E-18), as were secretion of norepinephrine (h^2^ = 66.5±5.0%, p = 3.2E-16) and dopamine (h^2^ = 56.5±5.6%, p = 1.8E-13), and eGFR displayed genetic co-determination (covariance) with norepinephrine (ρ_G_ = −0.557±0.088, p = 1.11E-08) as well as dopamine (ρ_G_ = −0.223±0.101, p = 2.3E-02). Since dopamine β-hydroxylase (DBH) catalyzes conversion of dopamine to norepinephrine, we studied functional variation at *DBH*; *DBH* promoter haplotypes predicted transcriptional activity (p<0.001), plasma DBH (p<0.0001) and norepinephrine (p = 0.0297) secretion; transcriptional activity was inversely (p<0.0001) associated with basal eGFR. Meta-analysis validated *DBH* haplotype effects on eGFR across 3 samples. In NIDDK-AASK, we established a role for *DBH* promoter variation in long-term renal decline rate (GFR slope, p = 0.003).

**Conclusions:**

The heritable GFR trait shares genetic determination with catecholamines, suggesting new pathophysiologic, diagnostic and therapeutic approaches towards disorders of GFR as well as CKD. Adrenergic activity may play a role in progressive renal decline, and genetic variation at *DBH* may assist in profiling subjects for rational preventive treatment.

## Introduction

The autonomic nervous system, and in particular its sympathetic branch, plays a role in physiological control of GFR as well as the development of CKD (chronic kidney disease) and eventually end-stage renal disease (ESRD) [1], [2]. Activation of sympathetic activity in CKD is characterized by increased muscle sympathetic nerve traffic [1] [3] and circulating levels of plasma norepinephrine [4]. Renal afferent sensory and efferent sympathetic innervation [5] may mediate the effect of chemoreceptors and baroreceptors in damaged kidneys [1] [3], [6], resulting in juxta-glomerular cell renin release, BP elevation, and acceleration of progressive renal dysfunction [7]. Indeed, agents that decrease sympathetic outflow have selective beneficial effects in progression of CKD, even at sub-antihypertensive doses [8], and renal sympathetic denervation is also an emerging therapy for intractable hypertension with progressive renal disease [9]. Sympathetic activation may also influence renal function by other means, including augmented renal vascular resistance (efferent or afferent arteriole), or increased tubular sodium reabsorption.

CKD (often defined as a chronic loss of GFR to <60 ml/min/1.73 m^2^) is an increasingly recognized syndrome, with substantial elevations in cardiovascular morbidity and mortality [10], [11]. CKD was responsible for the death of nearly 45,000 people in 2006, ranking as the ninth leading cause of death in the United States [12].

An improved understanding of the role of heredity in adrenergic control of GFR as well as progressive CKD may reveal novel pathways that could be exploited for preventive or even therapeutic strategies in CKD. Here we employed a twin pair design to explore the role of heredity in coupling of adrenergic and renal function, in healthy individuals as well as patients with progressive CKD.

## Results

### eGFR tertiles

#### Demographic traits

Table S1 in file S1 stratifies the twin/sibling study population by eGFR tertiles (using the CKD-EPI method) in those without CKD (eGFR>60 ml/min), as compared to those with CKD (eGFR≤60 ml/min). Demographic parameters differing by eGFR stratum included age (subsequently adjusted for), ethnicity and family history of hypertension.

#### Physical/physiological traits

SBP/DBP decreased in the higher eGFR tertiles (P<0.001), though the association disappeared once adjusted for age. BMI was slightly higher in the middle eGFR tertile.

#### Renal traits

Each estimator of GFR was significantly different by tertile in the age-adjusted model, as was eGFR between individuals with and without CKD (P<0.0001). Urine albumin excretion was elevated in subjects with CKD (p = 0.0164).

#### Adrenergic traits

**Figure 1:**
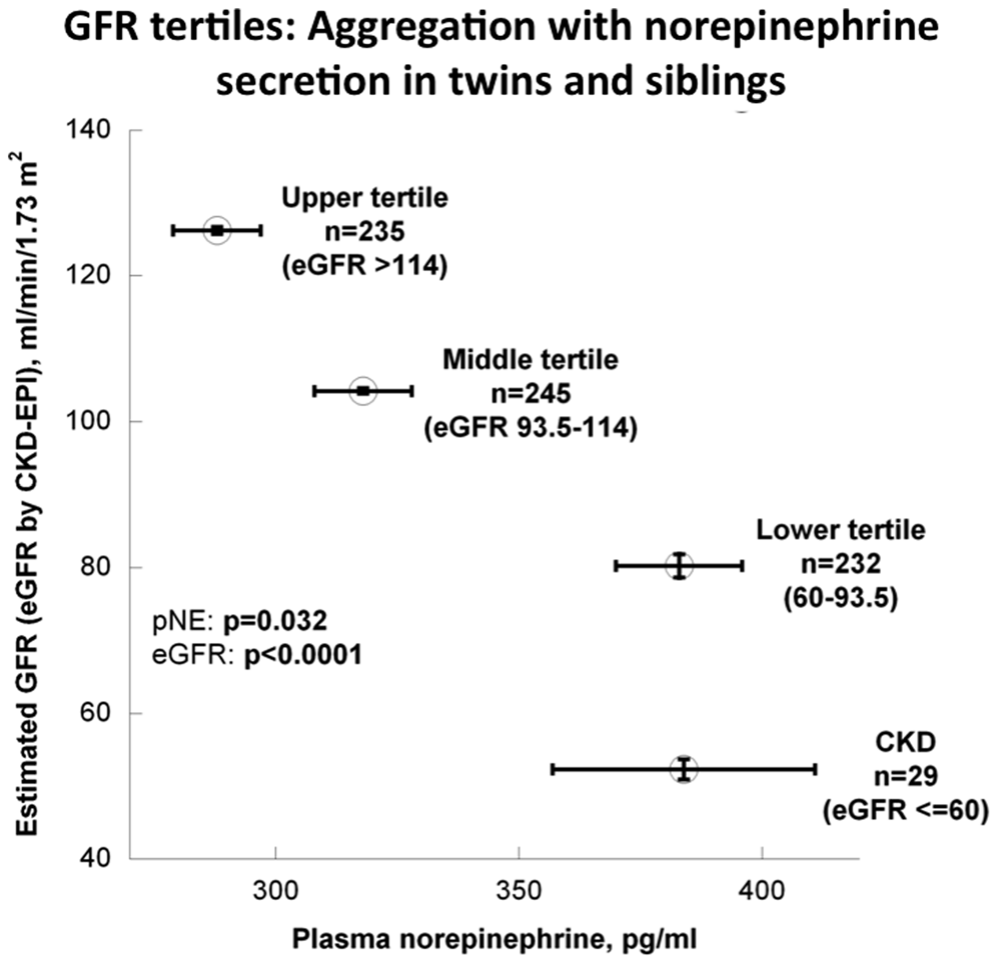
Adrenergic function and GFR: eGFR tertiles. Aggregation of eGFR tertiles with plasma norepinephrine in the entire study cohort, including those with CKD (eGFR≤60).

Individuals in the lower eGFR strata displayed higher plasma norepinephrine concentrations (Table S1 in file S1; Figure 1; p = 0.032), though other catecholamines were not different. To probe the relationship in individual detail, we found a significant inverse correlation between eGFR and plasma norepinephrine, whether tested in all subjects (ρ = −0.263, p<0.0001) or only in those without CKD (ρ = −0.266, p<0.0001) (Figure S1 in file S2).

#### eGFR trait-on-trait correlations and h^2^ in twins

**Figure 2:**
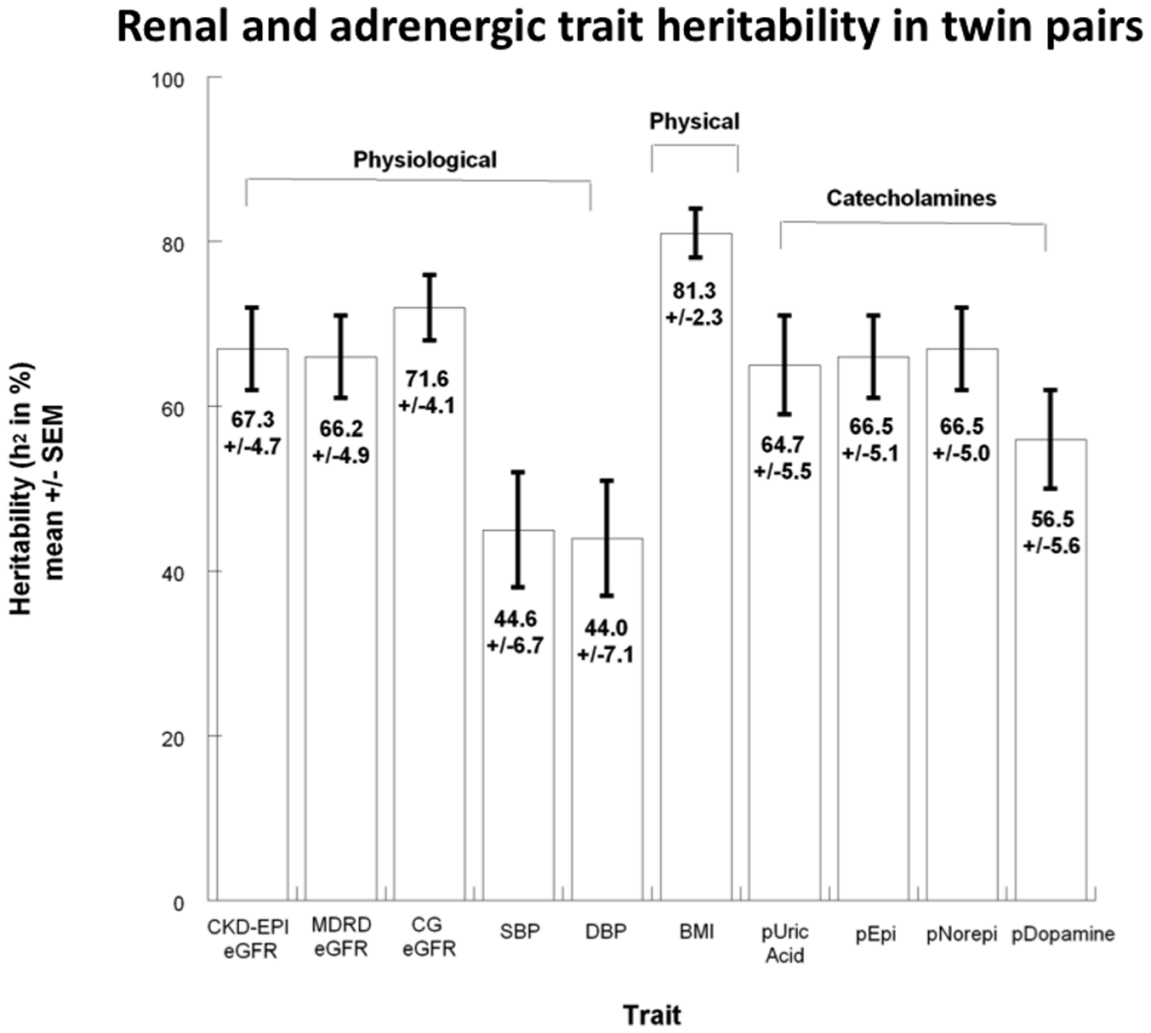
Heritability h^2^ and genetic covariance: Heritability (h^2^) estimates of CKD-EPI estimated glomerular filtration rate (eGFR) and renal, physiological, physical and catecholamine risk factors in the twin study cohort. The h^2^ estimates, expressed as % (± standard error of mean) of trait variance (h^2^ = V_G_/V_P_), obtained using SOLAR, suggest general agreement of the present cohort with other previously published research. h^2^ estimates arose from n = 340 (renal traits) to n = 357 (adrenergic traits) individuals. BMI indicates body mass index; SBP, systolic blood pressure; DBP, diastolic blood pressure; pEpi, plasma norepinephrine; pNorepi, plasma norepinephrine; pDopamine, plasma dopamine; CKD-EPI, Chronic Kidney Disease Epidemiology Collaboration formula; CG, Cockroft-Gault formula; MDRD, Modification of Diet in Renal Disease study formula.

Inter-individual correlations between eGFR and physiological or adrenergic traits are presented in Table S2 in file S1. As shown there and in Figure 2, heritability for eGFR was h^2^ = 67.0±5.0% (p<0.0001); heritability values for other physiological, physical and adrenergic traits are also displayed graphically in Figure 2. Heritability was highly significant for all adrenergic and renal traits: plasma norepinephrine (h^2^ = 66.5±5.0%, p<0.0001), plasma epinephrine (h^2^ = 66.5±5.1%, p<0.0001) and plasma dopamine (h^2^ = 56.5±5.6%, p<0.0001). Other physical traits such as BMI displayed an even higher rate of heritability, at h^2^ = 81.3±2.3%, p<0.0001.

#### Shared heritability in twins: Genetic covariance between eGFR and catecholamines

Several phenotypic traits that correlated with eGFR were analyzed for shared heredity (genetic covariance or pleiotropy) in twin pairs (Table S2 in file S1). As expected, CKD-EPI eGFR displayed substantial genetic covariance with MDRD eGFR (ρ_G_ = 0.98±0.008, p<0.0001) and Cockroft/Gault eGFR (ρ_G_ = 0.65±0.01, p<0.0001). Two catecholamine traits showed significant genetic covariance with eGFR: plasma norepinephrine (ρ_G_ = −0.56±0.09, p<0.0001) and plasma dopamine (ρ_G_ = −0.22±0.010, p<0.05). Figure S2 in file S2 depicts shared genetic (ρ_G_) versus environmental (ρ_E_) co-determination of eGFR with associated variables of interest, illustrating significant pleiotropy for eGFR with these two adrenergic traits.

### *DBH* functional genetic variation: Promoter haplotypes

#### *DBH* promoter transcription

**Figure 3:**
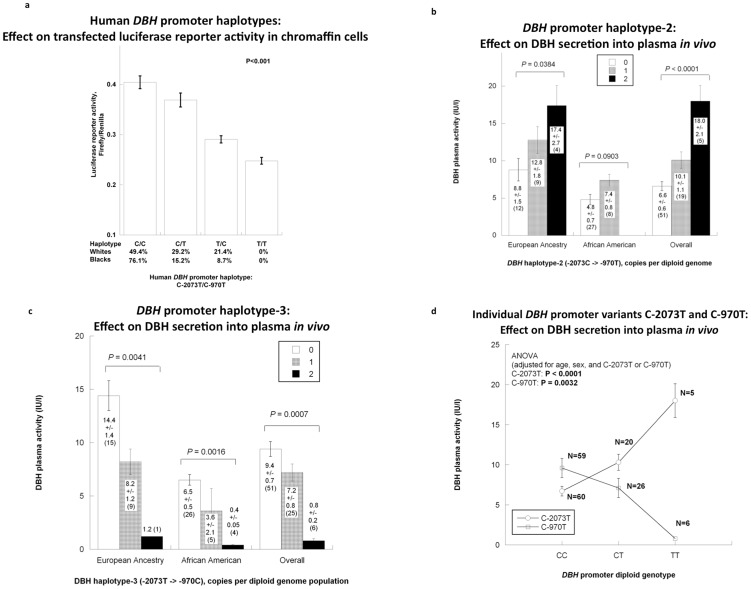
*DBH* promoter haplotypes (C-2073T→C-970T): Results for haplotype/luciferase reporter enzymatic activity in transfected chromaffin cells, as well as DBH secretion in humans. a. ***DBH*** **promoter haplotype expression in the nucleus**: Transcription in luciferase reporter plasmids transfected into chromaffin (PC12) cells. Each promoter transfection was done in 8 replicates. b. ***DBH*** **promoter haplotypes** ***in vivo*** **: Effects on plasma DBH activity**. Two functional promoter SNPs constituting a haplotype are shown in subjects of European ancestry, African American and the overall population. **Haplotype-2 (C→T)** is significantly associated with DBH activity in subjects from European ancestry as well as the overall study population. c. **Haplotype-3 (T→C)** is significantly associated with DBH activity in all groups. d. **Common promoter variants C-2073T and C-970T analyzed individually for effects on DBH secretion** ***in vivo***. Plasma DBH activity shows significant association with each of the common variants, both C-2073T and C-970T. To attain specificity, C-2073T or C-970T (as appropriate) were included as covariates, along with age and sex.

In reference to our previous work in the *DBH* promoter region [13], [14], there are two common functional variants that alter transcription of the gene: C-2073T (rs1989787) and C-970T (rs1611115), separated by only 1103 bp. We thus evaluated functional consequences of haplotypic variation at C-2073T→C-970T, using transfected promoter haplotype/luciferase reporter plasmids (Figure 3a), and found haplotype C→C to exhibit the highest gene expression, followed by C→T, then T→C, and finally T→T (note that T→T does not occur in human samples; Table S3 in file S1). Thus, haplotypes naturally occurring in humans (C→C, C→T, T→C) have pronounced effects on *DBH* transcription (p<0.001).

#### DBH secretion into plasma

Across biogeographic ancestries (white versus black), we found that DBH plasma activity substantially increased (p<0.0001) with increasing copy number (0,1,2 per genome) for haplotype-2 (C→T), with a directionally opposite effect (p = 0.0007) for haplotype-3 (T→C) (Figures 3b, 3c). We also found that the C→C haplotype-1 increased (p = 0.0124) plasma DBH activity (Figure S3 in file S1), but most prominently in African Americans, perhaps because of the relatively high frequency of haplotype C→C in that population (76.6% of chromosomes; Tables S3c and S4b in file S1). The theoretical T→T haplotype was not observed in any subject (0/458 chromosomes, Table S3c in file S1), reflecting the minor allele frequencies of T-alleles at both loci.

We explored directional effects for individual promoter SNP genotypes on DBH activity (Figure 3d): the minor (T) allele increased (p<0.0001) DBH activity at SNP C-2073T, while decreasing (p = 0.032) DBH activity at SNP C-970T.

#### Frequencies, LD, and biogeographic ancestry

Across biogeographic ancestry groups (Tables S3a and S3b in File S1), the two nearby promoter variants differed in allele and diploid genotype frequencies as well as extent of LD (linkage disequilibrium, as D′, r^2^, or chi^2^). Within the overall study population and specifically in the white ethnic subpopulation, we found minimal historical recombination as indicated by high LD values (p<0.0001; Table S3b in file S1). Based on this degree of LD, the experiment-wide significance threshold as determined by Nyholt's SNPSpD to maintain the type I (false positive) error rate at 5% or less is P≤0.026. However, greater historical recombination effects were reflected as reduced LD in the African-American (NIDDK-AASK) study population (Table S3b in file S1).

Of the 4 possible haplotypes across these two tightly linked *DBH* promoter variants in twin/siblings and AASK subjects, we imputed the existence of three common haplotypes: C→C, C→T, and T→C (Table S3c in file S1); theoretical haplotype T→T was not observed in at least 2n = 458 chromosomes (Tables S3c and S4a in file S1), consistent with the T-allele as the minor allele at both C-2073T and C-970T (Tables S3a and S3c in file S1).

#### *DBH* promoter functional genetic variation: Effects on renal and adrenergic traits

Since secretion of both norepinephrine and dopamine displayed pleiotropy with eGFR (Table S2 in File S1; Figure S2 in file S1), and DBH catalyzes the conversion of dopamine to norepinephrine, we also explored the effects of *DBH* genetic variation on eGFR and catecholamines.

#### Norepinephrine secretion

**Figure 4:**
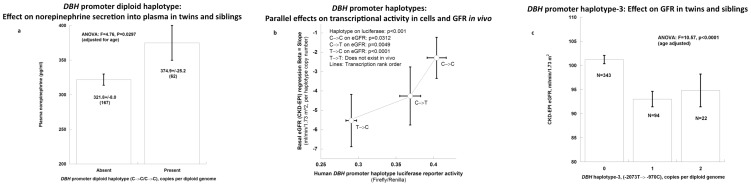
*DBH* promoter haplotype with adrenergic or GFR traits in twins and siblings. a. ***DBH*** **promoter diploid haplotype-1 (C→C) association with norepinephrine secretion**. b. ***DBH*** **promoter haplotypes**: Inverse association between transcriptional activity (transfected promoter/reporter plasmids in PC12 chromaffin cells) and eGFR. Each promoter transfection was done in 8 replicates. Haplotype numbers for this population were: C→C (n = 226; 29.4%), C→T (n = 134; %), T→C (n = 98; 21.4%), and T→T (n = 0). c. ***DBH*** **promoter haplotype-3 (T→C)**: Significant association (P<0.0001) with eGFR in an age-adjusted model.

Individuals homozygous for the most transcriptionally active (Figure 3a) promoter haplotype, C→C/C→C, displayed higher plasma norepinephrine concentration than all others (by ∼16%, p = 0.029; Table S4b in file S1; Figure 4a). In bivariate though not univariate models (Table S4a in file S1), promoter haplotypes also jointly influenced plasma norepinephrine and eGFR (C→C, p = 0.0075; T→C, p = 0.0022).

#### Basal eGFR

Overall, *DBH* promoter transcriptional activity displayed a clearly inverse relationship with basal eGFR (p<0.0001, Figure 4b). *DBH* promoter common haplotype-3 (T→C, across C-2073T→C-970T) displayed the most significant (P<0.0001; Table S4a in file S1) effect on eGFR, with the T→C haplotype diminishing the trait (Figure 4c). When results were tested in the largest biogeographic ancestry group (white, n = 229) alone, the effects persisted (p = 0.0377; Table S4c in file S1). *DBH* promoter diploid haplotypes also influenced eGFR (C→C/C→T, p = 0.0072; C→T/T→C, p = 0.0019; Table S4b in file S1). Individual SNP C-2073T predicted eGFR, though not norepinephrine (Table S4c in file S1). *DBH* promoter genetic variation did not influence urine albumin excretion in these subjects.

#### Extension of *DBH* promoter haplotype effects into additional population samples: Meta-analysis

Meta-analysis combining the twin/sibling pairs and two additional independent population samples (Kaiser-1 and Kaiser-2), for a total of n = 3063 subjects, indicated allelic effects consistent in magnitude (beta, or effect size per allele) and direction (sign on slope) across groups; the overall slope of the meta-analysis regression on haplotype-2 (C-2073→-970T, C→T) was significant, at beta (slope) = −1.321, with SE (of beta) = 0.560, and p = 0.018 (Table S5 in file S1). In a similar meta-analysis, haplotype T→C maintained marginal statistical significance on eGFR (p = 0.081).

#### *DBH* genetic variation and longitudinal progression of CKD (GFR slope) in the NIDDK-AASK trial

Very different *DBH* haplotype frequencies were observed in the African-American subjects as compared to other ethnicities in the twins/siblings (Table S3c in file S1; p = 0.0041), though not between blacks in the twin/sibling versus AASK (p = 0.99); especially prominent was the increased frequency of haplotype C→C in blacks (from 49.5% of chromosomes in whites, to 76.1% in AASK).

**Figure 5:**
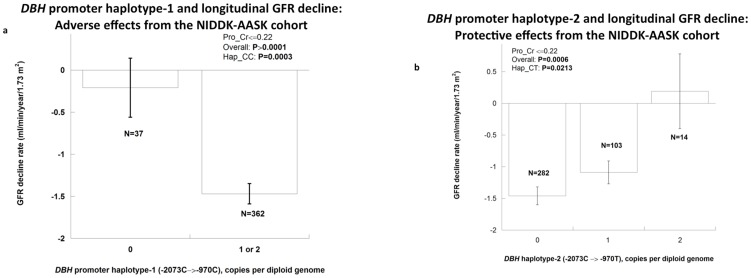
*DBH* promoter haplotype activity: Longitudinal effects on GFR decline in the NIDDK-AASK study population. GFRs were determined longitudinally by [^125^I]-iothalamate clearance. Subjects without excessive proteinuria at entry (urine protein/creatinine ratio ≤0.22 gm/gm at entry, N = 428) were analyzed. **a.** ***DBH*** **promoter haplotype-1 (C→C)** displaying a significant association (p = 0.0006) with GFR slope in an age-adjusted model. **b.** ***DBH*** **promoter haplotype-2 (C→T)** displaying a significant association (p = 0.0006) with GFR slope in an age-adjusted model.

*DBH* promoter haplotypes C→C and C→T displayed significant associations with GFR slope (Table S6a in file S1, each P<0.01). While the presence of haplotype C→C seemed to accelerate renal decline (p = 0.003, Figure 5a), haplotype C→T was protective, as a function of its copy number (0,1,2 copies/diploid genome; p = 0.0006, Figure 5b). At individual SNPs, *DBH* promoter variant C-970T retained significant association with GFR slope (Table S6b in file S1; p = 0.029). In the same individuals, *DBH* promoter genetic variation did not influence baseline (pre-study) urinary protein excretion (protein/creatinine ratio, mg/gm).

## Discussion

### Overview

**Figure 6:**
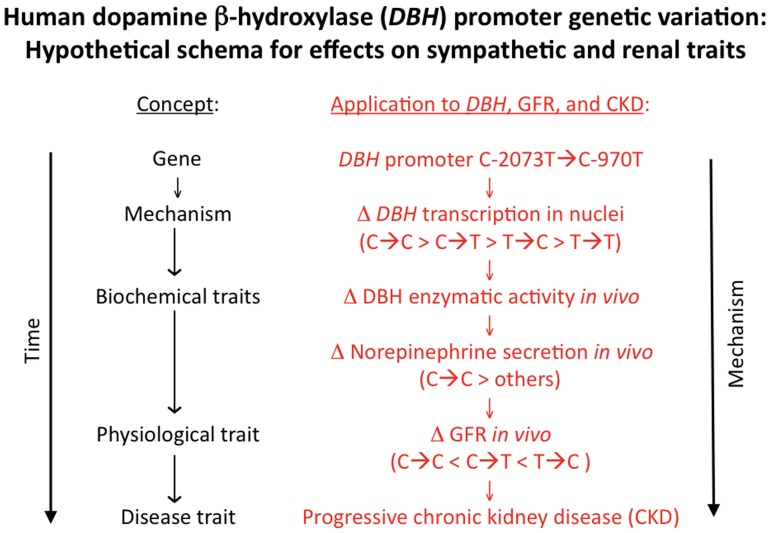
Human *DBH* genetic variation and its effects on sympathetic and renal traits: a schematic hypothesis. Framework for application of experimental results with *DBH* genetic variation at the promoter region: Alteration of *DBH* transcription and enzymatic activity affects norepinephrine, ultimately influencing GFR (physiological and disease traits).

Here we probed the aggregation of renal function with physical, physiological, and adrenergic traits, focusing on the role of heredity in control of GFR (as estimated with the CKD-EPI algorithm). We found plasma norepinephrine to be inversely associated with eGFR (Figure 1; Figure S1 in file S2) with an R^2^ (explanatory coefficient) of ∼6.5%, indicating that sympathetic over-activity is not restricted to End-Stage Renal Disease (ESRD) [15], but occurs in earlier stages of progressive renal failure [16]. As schematically depicted in Figure 6, sympathetic activation seems to play a fundamental role in control of renal function, which may contribute to progression of CKD. Indeed, other evidence suggests that decreased renal function mediated through sympathetic over-activation may contribute to arterial hypertension in humans [2] and experimental animals [8]. Other studies suggest a role for genetic variation in adrenergic receptor loci on progressive renal disease [17], [18].

### *DBH* functional genetic variation and renal function

Since norepinephrine is formed from dopamine in the catecholamine biosynthetic pathway through an enzymatic reaction catalyzed by *DBH*, and GFR displays genetic covariance with both norepinephrine and dopamine (Table S2 in File S1; Figure S2 in file S2), we targeted variation at the *DBH* gene for heritable effects on GFR. We began by looking at these two functional *DBH* promoter SNPs [13], [14] across four biogeographic ancestry groups (Tables S3b, S3c in file S1) and found that both SNPs are in close LD, especially in individuals of European ancestry.

Transcriptional activity of the *DBH* promoter variants was evaluated by transfection of promoter→luciferase reporter plasmids. Previously we identified two functional variants in the proximal human DBH promoter that alter transcription: C-970T [13] and C-2073T [14]. Transcriptional activity of the 4 haplotypes themselves ranked (Figure 3a): C→C>C→T>T→C>T→T. At C-970T, promoter activity ranked T>C allele [13], while at C-2073T the activity ranked C>T allele [14]. However, the individual allelic effects on transcription were background (i.e., haplotype) –specific [14]. A similar rank order of haplotype effects was established on DBH secretion *in vivo* (Figure 4a): C→C>C→T>T/C (note that the dual minor allele haplotype T→T was not observed in human samples).

In previous studies, we linked individual *DBH* promoter variants to DBH secretion as well as elevated blood pressure [13], [14]; since hypertension is coupled to progression of kidney dysfunction, we searched for *DBH* promoter haplotype effects on renal function, beginning with normal variation of eGFR in the healthy twin/sibling sample [17], [19]. In studies of basal (resting) eGFR, we found that each of the 3 *DBH* haplotypes – C→C (p = 0.0017), C→T (p = 0.0013), and T→C (p<0.0001) – displayed significant effects on the eGFR trait (Table S4a in file S1), and the overall effect was such that higher transcriptional activity paralleled basal eGFR (Figure 4b). Despite this overall picture, individual results on occasion deviated from expectation. For example, haplotype-3 (T→C) was associated with a decrease in not only plasma DBH (Figure 3c) but also eGFR (Figure 4c); here we cannot exclude reciprocal effects of central versus peripheral noradrenergic systems on sympathetic and renal traits [13] [20].

We extended the approach into progressive renal disease with the NIDDK-AASK longitudinal GFR decline (Table S5 in file S1; Figure 5); here the C→C haplotype, which is far more common in African Americans (at >76%, Table S3c in file S1), exerted a hazardous (steeper) effect on downward GFR slope (Figure 5a), while haplotype C→T seemed to exert a protective (flattening) effect on GFR slope (Figure 5b).

### Role of biogeographic ancestry and disease phenotype (GFR, basal versus change)

In order to minimize artificial marker on trait associations due to population admixture, we limited our genetic analyses on basal eGFR to people of European ancestry in the twin/sibling study (Figure 4), and to sub-Saharan ancestry in the NIDDK-AASK longitudinal cohort (Figure 5). Since the C-alleles at each SNP had the higher frequencies, it was perhaps not surprising to find effects of haplotype C→C on both basal eGFR (Figure 4b) and iothalamate GFR slope (Figure 5).

Why was the C→C haplotype associated with higher basal eGFR in healthy individuals (Figure 4b), but instead a risk haplotype for longitudinal renal decline in the NIDDK-AASK cohort (Figure 5a)? In accord with the “remnant nephron” hypothesis, initially higher GFR or “hyperfiltration”, while benign in healthy individuals (Figure 4b) may actually become a risk factor for more rapid decline of GFR in patients with of already damaged kidneys in CKD (Figure 5a) [21].

### Advantages and limitations of this study

Particular **strengths** of this study include: a) Well-characterized cohorts with multiple phenotypes profiled; b) Twin study design to allow genetic analyses such as heritability of GFR, as well as and genetic and environmental covariance for associated traits; c) Pleiotropic effects of *DBH* genetic variation on GFR and adrenergic traits; d) Novel effects of two functional *DBH* promoter variants on the GFR, shedding light on the adrenergic influence on renal function; and e) Extension of the findings into an African American cohort with progressive renal disease.

**Caveats** to interpretation of these results include the primarily white twin/sibling study sample, which may limit generalization of findings to other populations, particularly other ethnic groups; however, the relatively homogeneous biogeographic ancestry does limit the potential artifactual influence of admixture. In addition, our healthy study subjects (twins/siblings) were predominantly female, and the majority was recruited from southern California. Thus, the twin/sibling results might not be demographically generalizable to the population as a whole. Nonetheless, the NIDDK-AASK results do extend our findings into a minority (and disease) population. Heritability and genetic covariance results emerge from correlations, and it may not be possible to impute causality or directionality from such findings; such findings do however suggest hereditary control of physiological or disease pathways. ∼9% of our twin/sibling subjects reported antihypertensive drug treatment; vasoactive drugs may influence GFR, and this potential confounder is of uncertain significance here.

Finally, the phenotype of plasma norepinephrine steady-state concentration is not solely dependent on rate of norepinephrine biosynthesis or secretion; inter-individual variability in the steady-state concentration of plasma catecholamines can also be determined by removal processes including reuptake (by NET1 or OCT3) or metabolism/degradation (by COMT or MAOA/MAOB); indeed, when plasma norepinephrine is elevated in uremia, one causative factor seems to be diminished plasma clearance of the amine [22]. Why did we more frequently observe DBH haplotype predictions of eGFR than of plasma norepinephrine (Table S4a in file S1)? As noted above, plasma norepinephrine is only an imperfect index of sympathetic tone, and in addition its assay CV of ∼10% exceeds the 1.0–1.5% assay CV for creatinine, the basis of eGFR calculation.

After initial positive findings, we focused thereafter on the role of *DBH* genetic variation. During genome wide association studies (GWAS), other loci, such as *UMOD*, have been documented to have small but reproducible effects on eGFR [23]; here we took a different approach, focusing on a particular candidate pathway (Figure 6) suggested by the aggregation of adrenergic and renal traits in twin pairs (Table S2 in File S1; Figure S2 in file S2).

### Conclusions and perspectives

We conclude that GFR is a highly heritable trait that shares genetic determination with adrenergic phenotypes, in particular norepinephrine and dopamine secretion. Linking these observations (Figure 6), GFR is influenced by functional variation in the promoter region of the catecholamine biosynthetic enzyme *DBH*, and variants at the *DBH* locus seem to confer risk in progressive renal disease, and may assist in profiling appropriate subjects for rational preventive treatment. With this knowledge in hand, subjects with an initial decline in renal function could be profiled for risk of future accelerated decline. Furthermore, understanding the linkage between adrenergic (e.g., *DBH*) polymorphism and renal decline would suggest rational intervention with agents that interfere with the formation, release, or renal actions of the sympathetic neurotransmitter norepinephrine.

## Methods

### Twin and sibling study population and design (TSP)

Recruitment and characterization of the twin/sibling cohort has been previously described in detail [13], [24], [25]. With ongoing recruitment from a southern California twin registry and newspaper advertisement, the overall study consists of 208 male and 533 female subjects; ∼0.93% had a diagnosis of diabetes mellitus, while ∼4.8% had urine albumin excretion greater than the upper limit of normal (>30 mg albumin/gm creatinine). 237 twin pairs (from 237 nuclear families) were recruited, of which more than 75% (n = 178 pairs, 2n = 356 individuals; 238 monozygotic, 118 dizygotic) were self-identified as ethnically. Zygosity was both self-reported and further verified through extensive single nucleotide polymorphism (SNP) genotype analysis. Ethnicity was self-identified, where white ethnic groups were of European ancestry and African Americans of sub-Saharan ancestry. The study population had an average recorded age at the time of initial visit at 41.7±0.5 years. ∼9% reported drug treatment for hypertension. None of the participants had a history of renal failure. More information on the study cohort health status can be seen from previous work published from our laboratory, as well as Table S1 in file S1. Plasma DBH enzymatic activity (see below) was evaluated in an additional sample of n = 25 white and n = 35 African American individuals, none of whom displayed renal insufficiency.

### Approval

Human studies were approved by the UCSD HRPP (Human Research Protection Program, the name for our Institutional Review Board <http://irb.ucsd.edu/>), and subjects gave written informed consent.

### Phenotyping: Catecholamines, DBH enzymatic activity, and creatinine

Blood and urine samples were obtained after at least 3 hours of fasting. Seated individuals, with a heparin-lock intravenous cannula previously in place for 5 minutes, had blood drawn into EDTA (for catecholamines or creatinine) or heparin (for DBH activity) tubes. Anticoagulated blood was promptly chilled on ice (at 0°C) prior to centrifugation within 30 min for preparation of plasma, which was then frozen at −70°C prior to assays in batch. In untimed urine specimens, analytes were normalized to endogenous creatinine concentration in the same sample. Urine albumin excretion was measured as previously described [26] and normalized to creatinine in the same sample. A sensitive radioenzymatic assay was used to obtain plasma or urine catecholamine through the catechol-O-methylation process [4]. The catecholamine assay involved transfer of a ^3^H label to catecholamines (dopamine, norepinphrine, or epinephrine) from S-adenosylmethionine during O-methylation, mediated by the enzyme catechol-O-methyltransferase (COMT). Prior to O-methylation, plasma catecholamines were extracted into dilute acetic acid to remove COMT inhibitors in plasma. Assay sensitivities (lower limits of detection) were ∼10 pg for dopamine and norepinephrine, and ∼6 pg for epinephrine. Inter-assay coefficients of variation were ∼10% for urine dopamine, ∼20% for plasma dopamine, ∼10% for norepinephrine and ∼13% for epinephrine.

DBH enzymatic activity was measured in heparinized plasma by a modification of the spectrophotometric procedure [27], [28]. The synthetic DBH substrate tyramine is converted (in the presence of Cu^2+^, N-ethylmaleimide, and fumarate) to octopamine, which is then oxidized to parahydroxybenzaldehyde by sodium periodate; the oxidation is terminated by sodium metabisulfite, and the parahydroxybenzaldehyde product is then quantified by its absorbance at 330 nm. Results are expressed as international units per liter (IU/L) of plasma. The DBH activity inter-assay coefficient of variation was 4.5%.

An autoanalyzer (Beckman-Coulter; Brea, CA) measured levels of plasma and urine creatinine, by the Kinetic Jaffe Reaction with traceable isotope dilution-mass spectrometry [29], with a CV of 1.0–1.5%.

### Calculations

Algorithms to estimate glomerular filtration rate (GFR) included the Chronic Kidney Disease Epidemiology Collaboration (CKD-EPI) method, with a simplified equation [30]: eGFR = 141×min(sCr/k, 1)^a^×max(sCr/k, 1)^−1.209^×0.993^Age^×1.018 [if female]×1.159 [if black], where pCr is plasma creatinine (mg/dL), “k” is 0.7 for females and 0.9 for males, “a” is −0.329 for females and −0.411 for males, “min” indicates the minimum of sCr/k or 1, and “max” indicates the maximum of sCr/k or 1. Although the CKD-EPI formula is proposed to be superior to the Modification of Diet in Renal Disease Study (MDRD) and Cockcroft-Gault (C–G) equations in study populations including normal renal function, we estimated GFR by all three algorithms. Aside from the C–G formula, both CKD-EPI and MDRD formulas require plasma creatinine, age, sex and ethnicity. Creatinine was measured in plasma in the twin/sibling study, while serum creatinine was used in the Kaiser/replication study, and the NIDDK AASK study.

Body surface area (BSA, m^2^) was calculated from weight and height as measure of body size [31]. Body mass index (BMI, kg/m^2^) was obtained as a function of weight and height to measure body fat [32]. We normalized biochemical (urine epinephrine, norepinephrine, and dopamine) traits in urine to creatinine concentration in the same sample, to control for variation in urine flow rate (water excretion).

### Longitudinal significance of *DBH* promoter polymorphism in progressive renal disease: NIDDK-AASK trial

Subjects were recruited through a prospective trial involving 21 centers that has been previously described [33], [34]. Briefly, African Americans (by self-identification) with hypertension and hypertensive renal disease (entry GFR range 20–65 ml/min/1.73 m^2^ BSA, by [^125^I]-iothalamate clearance) were randomized to one of two blood pressure goal groups (“usual” mean arterial pressure goal of 102–107 mmHg, or to a lowered mean arterial pressure goal of ≤92 mmHg), and one of three blinded medication classes: ACE inhibition with ramipril; beta-adrenergic blockade with metoprolol; or calcium channel blockade with amlodipine. Since drugs such as ACE inhibitors influence GFR slope most prominently in patients without substantial proteinuria [35], analyses were limited to the 428 individuals, aged 21 to 71, with urine protein/creatinine ratio of ≤0.22 gm/gm at entry, and analyses were further adjusted for blood pressure goal and medication group. In the NIDDK-AASK trial, GFR was assessed by renal clearance of [^125^I]-iothalamate twice at baseline, then at 3 and 6 months from baseline, and then every 6 months to end of follow-up. Serum and urine creatinine and protein were measured by a central laboratory (Cleveland Clinic) at 6-month intervals. The primary AASK trial was completed before the inception of ClinicalTrials.gov. Availability of data and samples from the AASK trial is described at <https://www.niddkrepository.org/home/>.

### Human *DBH* promoter genomics and molecular biology

The human *DBH* promoter was re-sequenced as described for variant discovery, on an ABI-3100 capillary device, using Sanger-dideoxy chemistry and specific fluorescent base incorporation [13]. SNP polymorphisms (DBH promoter C-970T, rs1611115; and C-2073T, rs1989787) were scored in a two-stage assay [36]. In stage one, PCR primers flanking the polymorphism were used to amplify the target region from 5 ng of genomic DNA. In stage two, an oligonucleotide primer flanking the variant was annealed to the amplified template, and extended across the variant base. The mass of the extension product (wild-type versus variant) was scored by MALDI-TOF mass spectrometry (low mass allele versus high mass allele). The human *DBH* promoter was excised as a 3050-bp fragment by PCR from a large-insert (BAC) genomic clone spanning the entire *DBH* locus (clone RP11-317B10; from CHORI, at <http://bacpac.chori.org)>), and the 3050 bp promoter was subcloned into the polylinker (promoter insertion site) of a firefly luciferase reporter plasmid as described [13], followed by site-directed mutagenesis to create common haplotypes (verified by re-sequencing), and then transfected into PC12 chromaffin cells for assessment of promoter strength, normalizing firefly luciferase activity to Renilla luciferase from a co-transfected transfection efficiency control plasmid [13]. Each promoter transfection experiment was done in 8 replicates.

### Statistical analyses

Descriptive and inferential statistics were obtained at baseline from the entire study population using generalized estimating equations (GEE) implemented in SAS PROC GENMOD in order to account for intra-family correlations between/among twins, siblings and parents [37]. Tertiles of eGFR by CKD-EPI were classified by 33^rd^ and 67^th^ percentiles, used to compare traits of interest while adjusting for age as a covariate. GEE analysis tested for associations between *DBH* promoter variants or haplotypes with renal and adrenergic traits. Further inferential statistics through chi^2^ (or Fisher's Exact test) and logistic regression analysis provided information on distribution of haplotypes between/among ethnic groups and in determining the effect of *DBH* promoter genotype on eGFR. Univariate analyses through general linear models assessed the effect of *DBH* promoter haplotypes on eGFR and plasma norepinephrine.

In order to maintain the false positive (Type I error) rate at α<0.05 during genetic associations at a single locus, we used the Nyholt's single nucleotide polymorphism spectral decomposition (SNPSpD) correction to determined an experiment-wide significance threshold p-value [38], available at <http://gump.qimr.edu.au/general/daleN/SNPSpD/>. Haplotypes were imputed from unphased diploid genotypes on the *DBH* promoter region using an expectation-maximization algorithm in PLINK (version 1.07, Harvard University; Cambridge, MA), [39], available at <http://pngu.mgh.harvard.edu/~purcell/plink/>.

Heritability (h^2^) estimates of physiological and adrenergic traits were obtained through a variance component method in Sequential Oligogenic Linkage Analysis Routines (SOLAR) [40] available at <http://solar.txbiomedgenetics.org/download.html>. Heritability is estimated by the following equation: h^2^ = V_G_/V_P_, where V_G_ is additive genetic variance and V_P_ is total phenotypic variance. Through this method, a normal distribution in twin (monozygotic and dizygotic) pairs is assumed in order to maximize the likelihood with a mean dependent on traits of interest. Genetic covariance (or pleiotropy) of eGFR with other correlated heritable traits was estimated as ρ_G_ in SOLAR, while environmental covariance was estimated as parameter ρ_E_.

Analyses were conducted in SAS version 9.1 (Statistical Analysis System; Cary, NC), SPSS (IBM, Chicago, IL), Nyholt's SNPSpD <http://gump.qimr.edu.au/general/daleN/SNPSpD/>, Purcell's PLINK <http://pngu.mgh.harvard.edu/~purcell/plink/>, or SOLAR <http://solar.txbiomedgenetics.org/download.html>. Interested parties should contact the authors for further details on data and statistics in these studies.

### Replication and meta-analyses

Three observational study cohorts were examined, including not only the twins/siblings (previously described) but also two population samples from the Kaiser primary care Caucasian population whose medical information was obtained through routine health visits to Kaiser Permanente Medical Group, a primary healthcare organization in San Diego (CA, USA). Although previously described [41], the Kaiser study population from the first cohort included 1616 individuals and 735 individuals from the second cohort, providing us with a total sample size of 3063 subjects. Male and female subjects from this primary care organization were studied. Similar to the twin/sibling cohort, only individuals with eGFR by CKD-EPI>60 ml/min/1.73 m^2^ are included for analyses. Serum creatinine was determined in the Kaiser-Permanente clinical laboratory by spectrophotometric autoanalyzer (Jaffe reaction).

The genotype effect size (beta, or slope per allele), its SE (standard error) and p value were obtained by regression analysis in SPSS-17. Meta-analyses were carried out with the command META, testing fixed effect (i.e., genotype) models in STATA-12 (College Station, TX), after individual study regression analysis in SPSS, to derive significance as well as pooled genotype effect size (beta, or slope per allele) and its SE (standard error).

## Supporting Information

File S1 **Supporting tables.** Table S1. eGFR (by CKD-EPI): Stratification into tertiles. Descriptive and inferential statistics for the twin and sibling study population. Values are Mean ± (SEM), or (n%). Inferential statistics were derived from GEEs (PROC GENMOD in SAS) to account for correlations between twins. CKD-EPi eGFR is grouped by tertiles (formed through SAS using 33th and 67th percentile cut off points). Numbers (N) describe the number of individuals analyzed. As a control, we observed CKD-EPi eGFR trait based on CKD-EPI eGFR tertiles to be significant (P<0.0001). BMI indicates body mass index; BSA, body surface area; SBP, systolic blood pressure; DBP, diastolic blood pressure; CKD-EPI, Chronic Kidney Disease Epidemiology Collaboration formula; CG, Cockroft-Gault formula; MDRD, Modification of Diet in Renal Disease study formula. Significant differences (P<0.05) are bold. Table S2. Heritability (h^2^) as well as genetic and environmental covariance of eGFR. Heritability, shared genetic determination (genetic covariance, ρ_G_ also known as pleiotropy) and environmental determination (environmental covariance, ρ_E_) for traits correlated with CKD-EPI eGFR. ρ_G_ and ρ_E_ are fractions, scaled from −1 to +1, and determined in SOLAR. Pearson parametric trait on trait correlations is also reported. BMI indicates body mass index; eGFR, estimated glomerular filtration rate; SBP, systolic blood pressure; DBP, diastolic blood pressure; CKD-EPI, Chronic Kidney Disease Epidemiology Collaboration formula; CG, Cockroft-Gault formula; MDRD, Modification of Diet in Renal Disease study formula. Significant differences (*P*<0.05) are bold. Analysis was undertaken in MZ and DZ twin pairs of European ancestry. Table S3a. Frequencies of *DBH* promoter variant diploid genotypes across biogeographic ancestry groups. *Fisher's Exact Test p-value. Significant differences (*P*<0.05) are bold. Table S3b. LD (Linkage Disequilibrium) between *DBH* promoter variants C-2073T and C-970T in twin/sibling and NIDDK-AASK study populations. DBH, dopamine β-hydroxylase. * Fisher's Exact Test p-values. Significant differences (*P*<0.05) are bold. Table S3c. Frequency of *DBH* promoter alleles and haplotypes by ethnicity in the twin/sibling and NIDDK-AASK study populations. Allele and haplotype frequencies of *DBH* promoter variants in twin/sibling (N = 229, 2N = 458) and the NIDDK-AASK populations (N = 428, 2N = 856). Analysis is restricted to CKD-EPI eGFR>60 in twin/sibling population and with entry urine protein/creatinine ratio ≤0.22 gm/gm in NIDDK-AASK. Significant differences (*P*<0.05) are bold. Table S4a. *DBH* promoter haplotypes (C-2073T→C-970T): Renal and adrenergic trait associations in the twin/sibling population. Haplotypes are imputed from PLINK using the twin/sibling population (N = 229). Inferential and descriptive statistics between haplotypes and on trait were obtained through GEE. Significant differences (*P*<0.05) are bold. Haplotype T→T was not observed. Table S4b. *DBH* promoter diploid haplotype (C-2073T→C-970T) effects on renal and adrenergic traits in twins/siblings. One-Way ANOVA between DBH diploid haplotype and CKD-EPI eGFR and plasma norepinephrine in an age-adjusted model. P-values are age adjusted. Diploid genotype; No, 0 copies; Yes, 1 copy or more. Significant differences (*P*<0.05) are bold. Table S4c. Associations of renal and adrenergic traits with *DBH* promoter variant C-2073T in twins/siblings. DBH, dopamine β-hydroxylase. P-values are obtained through GEEs (PROC GENMOD). Significant differences (*P*<0.05) are bold. Table S5. Effect of *DBH* promoter haplotypes on eGFR: Extension to multiple independent groups, by meta-analysis. eGFR: Estimated glomerular filtration rate.CKD-EPI: Chronic Kidney Disease Epidemiology algorithm for GFR. KWE: Kaiser primary-care Caucasian population samples (cohorts 1&2). Table S6a. *DBH* promoter haplotype effects on progressive renal disease: Longitudinal GFR slope in the NIDDK-AASK cohort. One-Way ANOVA analysis of change in iothalamate GFR overtime with haplotypes adjusted for influential variables. Model I indicates adjustment for Pro/Cr, blood pressure (BP) goal, drug group, and mean baseline glomerular filtration rate (MB GFR). Model II indicates adjustment for Pro/Cr, BP goal, drug group, MB GFR, age at randomization and sex. DBH indicates dopamine β-hydroxylase. In this analysis, we focused on AASK individuals with entry urine protein/creatinine ratio ≤0.22 gm/gm (N = 428; see Methods). Significant differences (*P*<0.05) are bold. 2N = number of chromosomes. Table S6b. *DBH* SNP genotype effects on longitudinal GFR slope in the NIDDK-AASK population of African Americans with progressive renal disease. ANOVA analysis of change in GFR (by iothalamate clearance) over time adjusted for influential variables. Model-I indicates adjustment for Pro/Cr, blood pressure (BP) goal, drug group, and mean baseline glomerular filtration rate (MB GFR). Model-II indicates adjustment for Pro/Cr, BP goal, drug group, MB GFR, Age at randomization and sex. DBH indicates dopamine β-hydroxylase. In this analysis, we focused on AASK individuals with entry urine protein/creatinine ratio ≤0.22 gm/gm (N = 428; see Methods). Significant differences (*P*<0.05) are bold.
(DOC)

File S2 **Supporting figures.** Figure S1. Adrenergic function and GFR. Trait-on-trait correlation. Association of CKD-EPI eGFR with plasma norepinephrine in the segment of the cohort without CKD (i.e., eGFR>60). An inverse relationship is seen between plasma norepinephrine and estimated GFR by CKD-EPI. The inverse correlation was maintained when subjects with CKD were included. Figure S2. Heritability h^2^ and genetic covariance: Shared genetic and environmental co-determination for traits correlated with CKD-EPI eGFR. Covariance estimates (± SEM) are from analyses in SOLAR, and plotted with ρ_E_ as a function of ρ_G_, with a diagonal line of identity (Y = X). h^2^ estimates arose from n = 340 (renal traits) to n = 357 (adrenergic traits) individuals. P-values are given in Table S2 in File S1. Figure S3. *DBH* promoter haplotypes (C-2073T→C-970T): Results for DBH secretion in humans. Haplotype-1 (C→C) association with plasma DBH activity in African Americans, where C→C has a higher haplotype frequency (at ∼76% of chromosomes).
(PPTX)
