# Correction: Heritable Influence of DBH on Adrenergic and Renal Function: Twin and Disease Studies

**DOI:** 10.1371/journal.pone.0100351

**Published:** 2014-06-16

**Authors:** The PLOS ONE Staff

The first sentence of the sub-section “DBH secretion into plasma” of the Results is incorrect. The correct sentence is: Across biogeographic ancestries (white versus black), we found that DBH plasma activity substantially decreased with increasing copy number (0,1,2 per genome) for haplotype-2 (C→T), with a directionally opposite effect for haplotype-3 (T→C) (Figures 3b, 3c).

The last two sentences of the third paragraph of the “*DBH* functional genetic variation and renal function” sub-section of the Discussion should be deleted.

There are a number of errors in Figure 3. Please see the corrected Figure 3 here.

**Figure 3:**
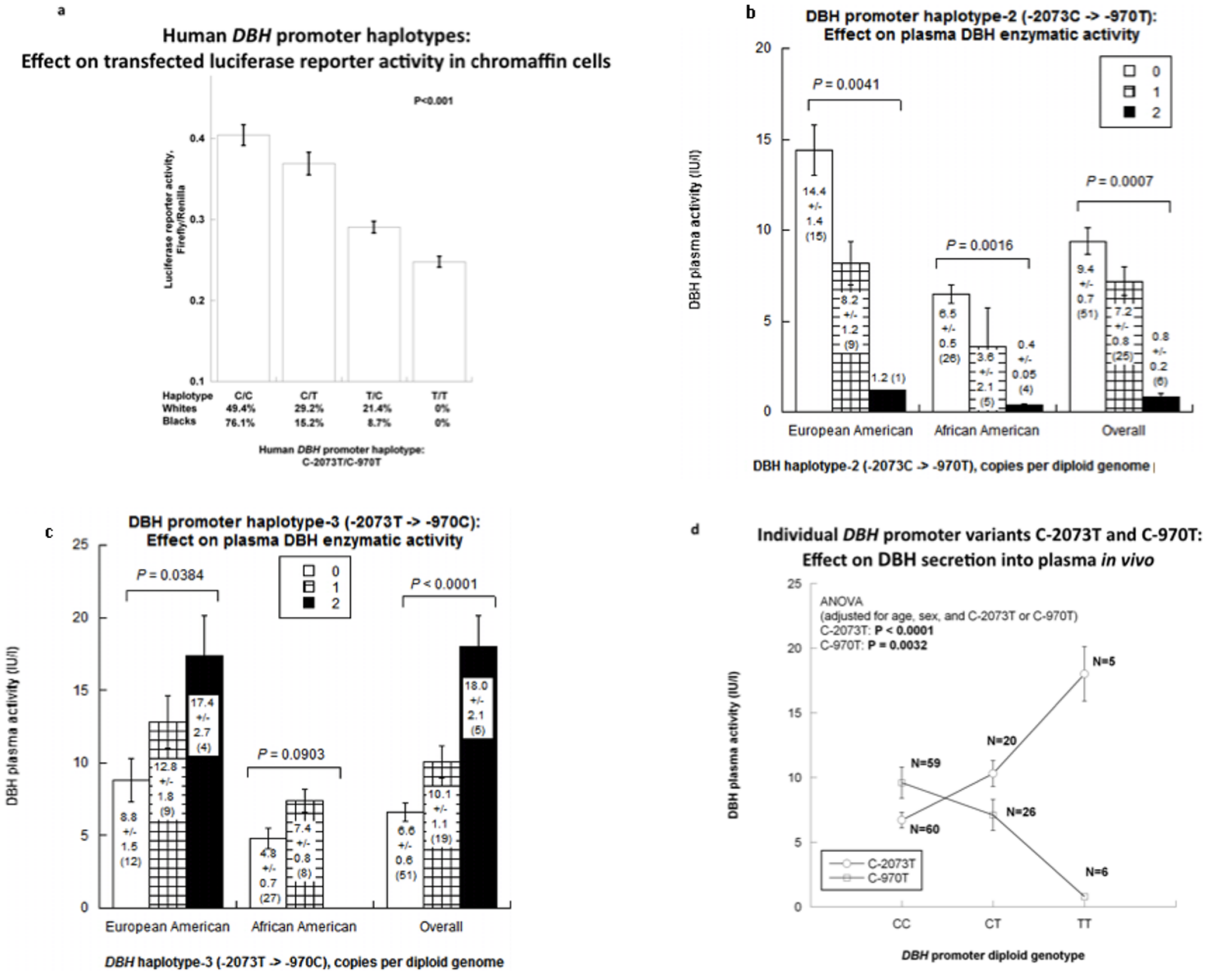
*DBH* promoter haplotypes (C-2073T→C-970T): Results for haplotype/luciferase reporter enzymatic activity in transfected chromaffin cells, as well as DBH secretion in humans. a. ***DBH*** **promoter haplotype expression in the nucleus**: Transcription in luciferase reporter plasmids transfected into chromaffin (PC12) cells. Each promoter transfection was done in 8 replicates. b. ***DBH*** **promoter haplotypes** ***in vivo***: **Effects on plasma DBH activity**. Two functional promoter SNPs constituting a haplotype are shown in subjects of European ancestry, African American and the overall population. **Haplotype-2 (C→T)** is significantly associated with DBH activity in subjects from European ancestry as well as the overall study population. c. **Haplotype-3 (T→C)** is significantly associated with DBH activity in all groups. d. **Common promoter variants C-2073T and C-970T analyzed individually for effects on DBH secretion** ***in vivo***. Plasma DBH activity shows significant association with each of the common variants, both C-2073T and C-970T. To attain specificity, C-2073T or C-970T (as appropriate) were included as covariates, along with age and sex.
