# Supplementary material for: Heritable Influence of DBH on Adrenergic and Renal Function: Twin and Disease Studies
Source: PLoS One. 2013 Dec 31;8(12):e82956. doi: 10.1371/journal.pone.0082956 (PMC3876991; doi:10.1371/journal.pone.0082956)
Supplement: File S1 — Supporting tables. Table S1. eGFR (by CKD-EPI): Stratification into tertiles. Descriptive and inferential statistics for the twin and sibling study population. Values are Mean ± (SEM), or (n%). Inferential statistics were derived from GEEs (PROC GENMOD in SAS) to account for correlations between twins. CKD-EPi eGFR is grouped by tertiles (formed through SAS using 33th and 67th percentile cut off points). Numbers (N) describe the number of individuals analyzed. As a control, we observed CKD-EPi eGFR trait based on CKD-EPI eGFR tertiles to be significant (P<0.0001). BMI indicates body mass index; BSA, body surface area; SBP, systolic blood pressure; DBP, diastolic blood pressure; CKD-EPI, Chronic Kidney Disease Epidemiology Collaboration formula; CG, Cockroft-Gault formula; MDRD, Modification of Diet in Renal Disease study formula. Significant differences (P<0.05) are bold. Table S2. Heritability (h2) as well as genetic and environmental covariance of eGFR. Heritability, shared genetic determination (genetic covariance, ρG also known as pleiotropy) and environmental determination (environmental covariance, ρE) for traits correlated with CKD-EPI eGFR. ρG and ρE are fractions, scaled from −1 to +1, and determined in SOLAR. Pearson parametric trait on trait correlations is also reported. BMI indicates body mass index; eGFR, estimated glomerular filtration rate; SBP, systolic blood pressure; DBP, diastolic blood pressure; CKD-EPI, Chronic Kidney Disease Epidemiology Collaboration formula; CG, Cockroft-Gault formula; MDRD, Modification of Diet in Renal Disease study formula. Significant differences (P<0.05) are bold. Analysis was undertaken in MZ and DZ twin pairs of European ancestry. Table S3a. Frequencies of DBH promoter variant diploid genotypes across biogeographic ancestry groups. *Fisher's Exact Test p-value. Significant differences (P<0.05) are bold. Table S3b. LD (Linkage Disequilibrium) between DBH promoter variants C-2073T and C-970T in twin/sibling and NIDDK-AA [file pone.0082956.s001.doc]

| **Supplementary Table S1. eGFR (by CKD-EPI): Stratification into tertiles.** | | | | | | | | | | |
| --- | --- | --- | --- | --- | --- | --- | --- | --- | --- | --- |
| **Variable** | **eGFR strata (by CKD-EPI, ml/min/1.73 m^2^)** | | | | | | | **Statistical comparisons** | | |
|  |  |  |  |  |  |  |  | **By tertiles (eGFR >60)** | | **CKD vs.**  **No CKD** |
|  | **All** | **CKD**  **(≤60)** | **Tertiles (in individuals with CKD-EPI eGFR >60 ml/min/1.73 m^2^)** | | | | | **Non-adjusted**  **(P-value)** | **Age-adjusted**  **(P-value)** | **Age-adjusted**  **(P-value)** |
|  |  |  | **<33^rd^ Percentile**  **(60-93.5)** | **33^rd^-67^th^ Percentile**  **(93.5-114)** | | **>67^th^ Percentile**  **(≥114)** | |  |  |  |
| **N** | 741 | 29 | 232 | | 245 | | 235 |  |  |  |
| **Demographics** |  |  |  | |  | |  |  |  |  |
| Age (years) | 41.7(0.5) | 57.1(2.2) | 48.1(1.1) | | 39.2 (0.8) | | 28.0(0.6) | **<0.0001** | **---** | --- |
| Sex (male/female) | 208(28%)/ 533(72%) | 10(34%)/ 19(66%) | 47(20%)/ 185(80%) | | 84(34%)/ 161(66%) | | 67(28%)/ 168(72%) | 0.1019 | 0.9032 | 0.3243 |
| Ethnicity (W/B/H/O) | 483(65%)/ 78(11%)/  66((9%)/ 114(15%) | 24(83%)/ 2(7%)/  2(7%)/ 1(3%) | 188(81%)/ 19(8%)/  11(5%)/ 14(6%) | | 156(64%)/ 22(9%)/  23(9%)/ 44(18%) | | 115(49%)/ 35(15%)/  30(13%)/ 55(23%) | 0.0753 | **<0.0001** | 0.2775 |
| Blood pressure status,  (high/normal) | 136(13%)/ 875(87%) | 24(83%)/ 5(17%) | 40(17%)/ 192(83%) | | 28(11%)/ 217(89%) | | 10(4%)/ 223(96%) | **<0.0001** | 0.1777 | 0.1647 |
| Family history of hypertension,  (yes/no/unknown) | 501(50%)/ 387(39%)/  109(11%) | 11(38%)/ 14(48%)/ 4(14%) | 118(51%)/ 87(38%)/  25(11%) | | 124(51%)/ 93(38%)/  28(11%) | | 141(60%)/ 80(34%)/ 13(6%) | **0.0210** | **0.0286** | 0.6589 |
| **Physical/Physiological** |  |  |  | |  | |  |  |  |  |
| Height (m) | 1.7(0.004) | 1.69(0.02) | 1.65(0.006) | | 1.68(0.007) | | 1.67(0.007) | 0.1578 | 0.1498 | **0.0138** |
| Weight (kg) | 72.9(0.69) | 74.2(3.3) | 71.2(1.1) | | 75.1(1.3) | | 72.3(1.2) | 0.6001 | **0.0252** | 0.8928 |
| BSA (m^2^) | 1.8(0.01) | 1.9(0.05) | 1.8(0.02) | | 1.9(0.02) | | 1.8(0.02) | 0.4875 | 0.1066 | 0.4958 |
| BMI (kg/m^2^) | 26.1(0.2) | 25.7(0.9) | 26.0(0.4) | | 26.5(0.4) | | 25.9(0.4) | 0.9001 | **0.0008** | 0.4958 |
| SBP (mmHg) | 124.1(0.5) | 128.0(2.7) | 126.2(1.0) | | 124.9(0.8) | | 120.8(0.9) | **0.0005** | 0.3608 | 0.4236 |
| DBP (mmHg) | 76.7(0.4) | 81.0(1.6) | 78.2(0.7) | | 77.5(0.6) | | 73.9(0.6) | **0.0002** | 0.4354 | 0.6490 |
| **Biochemical** |  |  |  | |  | |  |  |  |  |
| Plasma creatinine (mg/dL) | 0.81(0.01) | 1.3(0.05) | 0.92(0.01) | | 0.79(0.01) | | 0.66(.01) | **<0.0001** | **<0.0001** | **<0.0001** |
| eGFR by CKD-EPI (ml/min/1.73 m^2^) | 103.6(0.76) | 52.3(1.4) | 80.2(0.6) | | 104.2(0.4) | | 126.2(0.5) | **<0.0001** | **<0.0001** | **<0.0001** |
| eGFR by C-G (ml/min/1.73 m^2^) | 117.9(1.6) | 61.2(3.2) | 87.0(2.4) | | 120.2(2.1) | | 152.9(2.4) | **<0.0001** | **<0.0001** | **<0.0001** |
| eGFR by MDRD (ml/min/1.73 m^2^) | 114.0(1.2) | 58.5(1.8) | 86.5(1.4) | | 120.1(2.0) | | 153.4 (2.8) | **<0.0001** | **<0.0001** | **<0.0001** |
| Plasma active renin, pg/mL | 21.4(1.5) | 20.3(2.1) | 21.2(2.7) | | 21.2(2.7) | | 22.4(3.5) | 0.8907 | 0.9323 | 0.7297 |
| Urine Na+/creatinine, mEq/g | 150.6(5.1) | 134.5(21.0) | 112.5(7.7) | | 151.1(8.7) | | 189.6(10.1) | **<0.0001** | 0.1103 | 0.1588 |
| Urine K+/creatinine, mEq/g | 82.7(3.1) | 84.1(15.6) | 65.4(4.7) | | 86.1(5.7) | | 96.2(5.9) | **0.0002** | 0.1303 | 0.2166 |
| Urine Na+/K+ ratio | 2.3(0.05) | 2.0(0.3) | 2.1(0.1) | | 2.3(0.1) | | 2.5(0.1) | **0.0017** | 0.1044 | 0.8284 |
| **Catecholamines** |  |  |  | |  | |  |  |  |  |
| Plasma dopamine (pg/ml) | 26.4(5.1) | 25.4(4.4) | 41.9(16.1) | | 19.4(1.6) | | 18.5(1.7) | 0.1518 | 0.3790 | 0.2928 |
| Plasma epinephrine (pg/ml) | 28.1(0.8) | 26.3(5.4) | 28.2(1.5) | | 29.8(1.4) | | 26.3(1.2) | 0.3597 | 0.2942 | 0.6729 |
| Plasma norepinephrine (pg/ml) | 332.0(6.2) | 384.0(27.4) | 383.4(12.7) | | 318.0(10.3) | | 288.4(8.5) | **<0.0001** | **0.0318** | 0.9288 |
| Urine dopamine/Cr, pg/mg | 254366  (14813) | 200748  (38942) | 207636  (33440) | | 226891  (18205) | | 337365  (25023) | **0.0032** | 0.0559 | 0.6063 |
| Urine epinephrine/Cr, pg/mg | 17845  (985) | 27086  (9644) | 14623  (1696) | | 17580  (1612) | | 20065  (1473) | **0.0367** | 0.4733 | 0.1089 |
| Urine norepinephrine/Cr, pg/mg | 40864  (2076) | 40879  (8670) | 33717  (3608) | | 38645  (3490) | | 50354  (3910) | **0.0025** | 0.0691 | 0.3896 |

Descriptive and inferential statistics for the twin and sibling study population. Values are Mean±(SEM), or (n%) . Inferential statistics were derived from GEEs (PROC GENMOD in SAS) to account for correlations between twins. CKD-EPi eGFR is grouped by tertiles (formed through SAS using 33^th^ and 67^th^ percentile cut off points). Numbers (N) describe the number of individuals analyzed. As a control, we observed CKD-EPi eGFR trait based on CKD-EPI eGFR tertiles to be significant (*P*<0.0001). BMI indicates body mass index; BSA, body surface area; SBP, systolic blood pressure; DBP, diastolic blood pressure; CKD-EPI, Chronic Kidney Disease Epidemiology Collaboration formula; CG, Cockroft-Gault formula; MDRD, Modification of Diet in Renal Disease study formula. Significant differences (*P*<0.05) are **bold**.

|  | | | | | | | | | | | |  |
| --- | --- | --- | --- | --- | --- | --- | --- | --- | --- | --- | --- | --- |
| **Supplementary Table S2. Heritability (h^2^) as well as genetic and environmental covariance of eGFR.** | | | | | | | | | | | |  |
| **Phenotype** | **Heritability** | | | **Correlation with eGFR**  **(by CKD-EPI)** | | **Genetic co-determination**  **(with CKD-EPI)** | | | **Environmental co-determination**  **(with CKD-EPI)** | | | **N** |
|  | **h^2^** | **SEM** | **P-value** | **ρ** | **P-value** | **ρ_G_** | **SEM** | **P-value** | **ρ_E_** | **SEM** | **P-value** |  |
| **Physiological** |  |  |  |  |  |  |  |  |  |  |  |  |
| eGFR by CKD-EPI | 0.673 | 0.0473 | **3.80E-18** | **-** | **-** | **-** | **-** | **-** | **-** | **-** | **-** | 340 |
| eGFR by MDRD | 0.662 | 0.0483 | **3.59E-17** | 0.8399 | **<0.0001** | 0.984 | 0.0084 | **5.53E-19** | 0.850 | 0.0245 | **2.98E-64** | 340 |
| eGFR by C-G | 0.716 | 0.0411 | **3.16E-22** | 0.7042 | **<0.0001** | 0.653 | 0.1020 | **2.18E-13** | 0.644 | 0.0528 | **1.91E-16** | 346 |
| SBP | 0.446 | 0.0669 | **1.60E-08** | -0.1892 | **<0.0001** | 0.096 | 0.1147 | 0.408 | 0.103 | 0.0902 | 0.256 | 386 |
| DBP | 0.440 | 0.0707 | **1.0E-07** | -0.2116 | **<0.0001** | -0.045 | 0.1196 | 0.708 | 0.061 | 0.0926 | 0.510 | 386 |
| **Physical** |  |  |  |  |  |  |  |  |  |  |  |  |
| BMI | 0.813 | 0.0278 | **3.67E-33** | -0.0115 | 0.7553 | 0.045 | 0.0885 | 0.609 | 0.027 | 0.0911 | 0.765 | 387 |
| **Catecholamines** (plasma) |  |  |  |  |  |  |  |  |  |  |  |  |
| Epinephrine | 0.665 | 0.0509 | **5.1E-16** | -0.0217 | 0.5729 | -0.088 | 0.1031 | 0.393 | 0.126 | 0.0926 | 0.178 | 357 |
| Norepinephrine | 0.665 | 0.0503 | **3.2E-16** | -0.2633 | **<0.0001** | -0.557 | 0.0879 | **1.11E-08** | 0.073 | 0.0893 | 0.417 | 359 |
| Dopamine | 0.565 | 0.0562 | **1.8E-13** | -0.0766 | **0.0463** | -0.223 | 0.1009 | **0.0298** | 0.084 | 0.0886 | 0.345 | 357 |

Heritability, shared genetic determination (genetic covariance, ρ_G_ also known as pleiotropy) and environmental determination (environmental covariance, ρ_E_) for traits correlated with CKD-EPI eGFR. ρ_G_ and ρ_E_ are fractions, scaled from -1 to +1, and determined in SOLAR. Pearson parametric trait on trait correlations is also reported. BMI indicates body mass index; eGFR, estimated glomerular filtration rate; SBP, systolic blood pressure; DBP, diastolic blood pressure; CKD-EPI, Chronic Kidney Disease Epidemiology Collaboration formula; CG, Cockroft-Gault formula; MDRD, Modification of Diet in Renal Disease study formula. Significant differences (*P*<0.05) are **bold**. Analysis was undertaken in MZ and DZ twin pairs of European ancestry.

| **Supplementary Table S3a. Frequencies of *DBH* promoter variant diploid genotypes across biogeographic ancestry groups.** | | | | | | | | |
| --- | --- | --- | --- | --- | --- | --- | --- | --- |
| **Ethnicity** | **C-2073T (N=353)** | | | | **C-970T (N=350)** | | | |
|  | **C/C** | **C/T** | **T/T** | **P-value*** | **C/C** | **C/T** | **T/T** | **P-value*** |
| White (n=249) | 126(51%) | 99(40%) | 24(9%) | **<0.0001** | 163(65%) | 67(27%) | 19(8%) | 0.6827 |
| Black (n=24) | 19(80%) | 4(16%) | 1(4%) |  | 18(78%) | 5(22%) | 0(0%) |  |
| Hispanic (n=19) | 10(53%) | 7(37%) | 2(10%) |  | 13(68%) | 4(21%) | 2(11%) |  |
| Other (n=61) | 49(80%) | 12(20%) | 0(0%) |  | 35(59%) | 19(32%) | 5(9% |  |
| *Fisher’s Exact Test p-value. Significant differences (*P*<0.05) are **bold**. | | | | | | | | |

| **Supplementary Table S3b. LD (Linkage Disequilibrium) between *DBH* promoter variants C-2073T and C-970T in twin/sibling and NIDDK-AASK study populations.** | | | | | |
| --- | --- | --- | --- | --- | --- |
| **Population** | **N (individuals)** | **D’** | **R^2^** | **χ2** | **P-value** |
| All | 329 | 0.99653 | 0.092 | 34.5 | **0.000001** |
| White | 229 | 0.99874 | 0.112 | 33.3 | **0.000001** |
| Black | 22 | 0.66008 | 0.008 | --- | 0.9990* |
| Hispanic | 19 | 0.95578 | 0.109 | --- | 0.4737* |
| Other | 59 | 0.97875 | 0.037 | --- | 0.5461* |
| Black (AASK) | 428 | 0.96050 | 0.016 | --- | 0.1732* |
| DBH, dopamine β-hydroxylase. * Fisher’s Exact Test p-values. Significant differences (*P*<0.05) are **bold**. | | | | | |

| **Supplementary Table S3c. Frequency of *DBH* promoter alleles and haplotypes by ethnicity in the twin/sibling and NIDDK-AASK study populations.** | | | | | | |
| --- | --- | --- | --- | --- | --- | --- |
| **Population (**n individuals) | **Minor (T) allele frequency** | | **Haplotypes across C970T → C2073T, frequencies (2N, %)** | | | |
|  | C970**T**  rs1611115 | C2073**T**  rs1989787 | **C→C**  **2H-1** | **C→T**  **2H-2** | **T→C**  **2H-3** | **T→T**  **2H-4** |
| White (TSP, n=229) | 0.2108 | 0.2952 | 226, 49.4% | 134, 29.2% | 98, 21.4% | 0% |
| Black (TSP, n=22) | 0.125 | 0.1087 | 34, 76.6% | 6, 12.6% | 4, 10.4% | 0% |
| Hispanic (TSP, n=19) | 0.2105 | 0.2895 | 19, 50.0% | 8, 21.1% | 11, 28.9% | 0% |
| Other (TSP (n=59)) | 0.2458 | 0.09836 | 77, 65.3% | 29, 24.6% | 12, 10.2% | 0% |
| Black (AASK (n=428)) | 0.1492 | 0.08382 | 648, 76.1% | 135, 15.2% | 73, 8.7% | 0% |
| TSP only (across ethnicities)  χ^2^, p | χ^2^=3.37, p=0.338 | χ^2^=25.24, **p<0.0001** | χ^2^=24.14, **p=0.0041** | | | |
| Black (TSP vs AASK)  χ^2^, p | χ^2^=0.29, p=0.5902 | χ^2^=0.76, p=0.3833 | χ^2^=0.08, p=0.9941 | | | |

Allele and haplotype frequencies of *DBH* promoter variants in twin/sibling (N=229, 2N=458) and the NIDDK-AASK populations (N=428, 2N=856). Analysis is restricted to CKD-EPI eGFR >60 in twin/sibling population and with entry urine protein/creatinine ratio ≤0.22 gm/gm in NIDDK-AASK. Significant differences (*P*<0.05) are **bold**.

| **Supplementary Table S4a. *DBH* promoter haplotypes (C-2073T → C-970T): Renal and adrenergic trait associations in the twin/sibling population.** | | | | | | |
| --- | --- | --- | --- | --- | --- | --- |
| **Haplotype number** | **CKD-EPI eGFR (>60)** | | **Plasma norepinephrine** | | **Bivariate (eGFR plus norepinephrine) analysis** | |
|  | **F** | **P-value** | **F** | **P-value** | **F** | **P-value** |
| 2H-1 (C→C) | 6.48 | **0.0017** | 1.72 | 0.4238 | 3.51 | **0.0075** |
| 2H-2 (C→T) | 6.78 | **0.0013** | 1.84 | 0.3981 | 2.36 | 0.0518 |
| 2H-3 (T→C) | 10.57 | **<0.0001** | 1.08 | 0.5826 | 4.21 | **0.0022** |
|  |  |  |  |  |  |  |

Haplotypes are imputed from PLINK using the twin/sibling population (N=229). Inferential and descriptive statistics between haplotypes and on trait were obtained through GEE. Significant differences (*P*<0.05) are **bold**. Haplotype T🡪T was not observed.

| **Supplementary Table S4b. *DBH* promoter diploid haplotype (C-2073T → C-970T) effects on renal and adrenergic traits in twins/siblings.** | | | | | | | |
| --- | --- | --- | --- | --- | --- | --- | --- |
| **Diploid haplotype** | **Frequency, N (%)** | | | **CKD-EPI eGFR (>60)** | | **Plasma norepinephrine** | |
|  | **Absent** | | **Present** | **F** | **P-value** | **F** | **P-value** |
| C→C/C→C | 165 (72%) | | 64 (28%) | 0.02 | 0.8756 | 4.76 | **0.0297** |
| C→C/C→T | 161(70%) | 68 (30%) | | 7.27 | **0.0073** | 1.94 | 0.1644 |
| C→C/T→C | 198(86%) | 31 (14%) | | 3.35 | 0.0679 | 1.51 | 0.2202 |
| C→T/T→C | 203(89%) | 26 (11%) | | 9.78 | **0.0019** | 0.45 | 0.5023 |
| C→T/C→T | 210 (92%) | 19 (8%) | | 0.29 | 0.5936 | 0.59 | 0.4418 |
| T→C/T→C | 208 (91%) | 21 (9%) | | 1.72 | 0.1908 | 0.05 | 0.8149 |
| C→C/T→T | 229(100%) | 0(0%) | | - | - | - | - |
| C→T/T→T | 229(100%) | 0(0%) | | - | - | - | - |
| T→C/T→T | 229(100%) | 0(0%) | | **-** | **-** | **-** | **-** |
| T→T/T→T | 229(100%) | 0(0%) | | **-** | **-** | **-** | **-** |

One-Way ANOVA between DBH diploid haplotype and CKD-EPI eGFR and plasma norepinephrine in an age-adjusted model. P-values are age adjusted. Diploid genotype; No, 0 copies; Yes, 1 copy or more. Significant differences (*P*<0.05) are **bold**.

| **Supplementary Table S4c. Associations of renal and adrenergic traits with *DBH* promoter variant C-2073T in twins/siblings.** | | | |
| --- | --- | --- | --- |
|  |  | **Dependent variable** | |
| **Independent variable:**  **C-2073T** | **N (individuals)** | **eGFR (by CKD-EPI,**  **>60 ml/min/1.73 m^2^)** | **Plasma norepinephrine, pg/ml** |
|  |  | **P-value** | **P-value** |
| Adjusted for age | 353 | **0.0024** | 0.3292 |
| Adjusted for age + ethnicity | 353 | 0.0607 | 0.2751 |
| Adjusted for age (white only) | 229 | **0.0377** | 0.2925 |
| Adjusted for age (black only) | 24 | 0.2903 | 0.1624 |
| DBH, dopamine β-hydroxylase. P-values are obtained through GEEs (PROC GENMOD). Significant differences (*P*<0.05) are **bold**. | | | |

| **Supplementary Table S5. Effect of *DBH* promoter haplotypes on eGFR: Extension to multiple independent groups, by meta-analysis.** | | | | | | | | | | |
| --- | --- | --- | --- | --- | --- | --- | --- | --- | --- | --- |
| **Group** | ***DBH* promoter haplotype** | **Haplotype frequency** | **N** | **Trait** | **Regression model** | **Meta-analysis model** | **Beta**  **(allele slope)** | **SE (of beta)** | **P-value** |  |
| Twins/  sibilings | C→T | 21.4% | 712 | CKD-EPI eGFR | Additive (0,1,2), age-adjusted |  | -4.258 | 1.504 | - |  |
| KWE-1 | C→T | 21.1% | 1616 | CKD-EPI eGFR | Additive (0,1,2), age-adjusted |  | -1.529 | 0.821 | - |  |
| KWE-2 | C→T | 21.1% | 735 | CKD-EPI eGFR | Additive (0,1,2), age-adjusted |  | -0.053 | 0.889 | - |  |
| **Meta-result** | **C→T** |  | **3063** |  |  | **Fixed effects** | **-1.321** | **0.560** | **0.018** |  |
|  |  |  |  |  |  |  |  |  |  |  |
| Twins/  sibilings | T→C | 29.2% | 712 | CKD-EPI eGFR | Additive (0,1,2), age-adjusted |  | -5.527 | 1.350 | - |  |
| KWE-1 | T→C | 34.6% | 1616 | CKD-EPI eGFR | Additive (0,1,2), age-adjusted |  | 0.858 | 0.687 | - |  |
| KWE-2 | T→C | 33.0% | 735 | CKD-EPI eGFR | Additive (0,1,2), age-adjusted |  | -1.371 | 0.743 | - |  |
| **Meta-result** | **T→C** |  | **3063** |  |  | **Fixed effects** | **-0.825** | **0.472** | **0.081** |  |
| eGFR: Estimated glomerular filtration rate.  CKD-EPI: Chronic Kidney Disease Epidemiology algorithm for GFR.  KWE: Kaiser primary-care Caucasian population samples (cohorts 1&2). | | | | | | | | | |  |

| **Supplementary Table S6a. *DBH* promoter haplotype effects on progressive renal disease:**  **Longitudinal GFR slope in the NIDDK-AASK cohort.** | | | |
| --- | --- | --- | --- |
| **Haplotype number** | **Frequency, 2N (%)** | **Effect on GFR slope (P-value)** | |
|  |  | **Model-I** | **Model-II** |
| 2H-1 (C→C) | 648 (76.1%) | **0.0017** | **0.0015** |
| 2H-2 (C→T) | 135 (15.2%) | **0.0109** | **0.0124** |
| 2H-3 (T→C) | 73 (8.7%) | 0.3303 | 0.3119 |
| 2H-4 (T→T) | 0 (0%) | - | - |

One-Way ANOVA analysis of change in iothalamate GFR overtime with haplotypes adjusted for influential variables. Model I indicates adjustment for Pro/Cr, blood pressure (BP) goal, drug group, and mean baseline glomerular filtration rate (MB GFR). Model II indicates adjustment for Pro/Cr, BP goal, drug group, MB GFR, age at randomization and sex. DBH indicates dopamine β-hydroxylase. In this analysis, we focused on AASK individuals with entry urine protein/creatinine ratio ≤0.22 gm/gm (N=428; see Methods). Significant differences (*P*<0.05) are **bold**. 2N = number of chromosomes.

| **Supplementary Table S6b. *DBH* SNP genotype effects on longitudinal GFR slope in the NIDDK-AASK population of African Americans with progressive renal disease.** | | |
| --- | --- | --- |
| **Independent variable** | **Effect on GFR slope (P-value)** | |
|  | **Model-I** | **Model-II** |
| DBH promoter C-970T | **0.0290** | **0.0352** |
| DBH promoter C-2073T | 0.2906 | 0.2785 |
| ANOVA analysis of change in GFR (by iothalamate clearance) over time adjusted for influential variables. Model-I indicates adjustment for Pro/Cr, blood pressure (BP) goal, drug group, and mean baseline glomerular filtration rate (MB GFR). Model-II indicates adjustment for Pro/Cr, BP goal, drug group, MB GFR, Age at randomization and sex. DBH indicates dopamine β-hydroxylase. In this analysis, we focused on AASK individuals with entry urine protein/creatinine ratio ≤0.22 gm/gm (N=428; see Methods). Significant differences (*P*<0.05) are **bold**. | | |
